# Optimizing micafungin dosing in critically ill patients: what about extracorporeal therapies?

**DOI:** 10.1186/s13054-018-2231-6

**Published:** 2018-11-01

**Authors:** Patrick M. Honore, David De Bels, Leonel Barreto Gutierrez, Sebastien Redant, Rachid Attou, Andrea Gallerani, Herbert D. Spapen

**Affiliations:** 1 ICU Department, Centre Hospitalier Universitaire Brugmann, Place Van Gehuchtenplein, 4, 1020 Brussels, Belgium; 2 Universitair Ziekenhuis Brussel, VUB University, Brussels, Belgium

We applaud the recent work of Maseda et al. [1] and the additional comments of Agrifoglio et al. [2] which offer insight into optimizing micafungin treatment in obese patients and in patients with burn injury. We would like to draw attention to another group of critically ill patients who might benefit from dose adaptation of micafungin, i.e., those undergoing continuous renal replacement therapy (CRRT) and extracorporeal membrane oxygenation (ECMO).

Being highly (> 90%) protein-bound, echinocandins are unlikely to be removed by hemodialysis or hemofiltration through convection. Standard dosing thus is considered to be appropriate during CRRT. However, drug adsorption on the filter membrane should not be underestimated. Adsorption mainly depends on electrical charge of the membrane, pH, and membrane surface area. The increasingly used polyacrylonitrile membranes (e.g., the acrylonitrile 69 (AN69) membrane) have the highest adsorption capacity. Pharmacokinetics (PK) of caspofungin and anidulafungin are barely influenced by adsorption, probably because both drugs are given as a loading dose to rapidly reach steady-state concentrations. Micafungin, however, is not administered as a loading dose, which may result in lower plasma levels during the first days of treatment. Vossen et al. [3] reported a higher clearance of micafungin in patients undergoing continuous veno-venous hemodiafiltration with modified AN69 membranes than in patients undergoing continuous hemodialysis with polysulfone membranes. Although maximum serum drug concentrations were higher than in the Maseda study, a 100 mg micafungin dose failed to achieve PK targets in patients with more resistant candida strains [3].

The main mechanisms that determine drug PK during ECMO are sequestration in the circuit, increased distribution volume, and membrane adsorption. In contrast with other echinocandins, micafungin is extensively extracted during ECMO. Micafungin plasma levels at 24 h are significantly reduced, regardless of ECMO circuit configuration or presence of a hemofilter [4]. ECMO was found to reduce the serum micafungin concentration, and consequently the area under the curve, by 23% in critically ill patients [5].

Taken together, higher doses of micafungin are recommended in patients receiving CRRT (150 to 200 mg daily) or ECMO (at least 200 mg daily). More PK studies are warranted to assure whether the proposed dose augmentation results in therapeutically relevant plasma levels, especially when confronted with more resistant *Candida* species.

## Abbreviations

AN 69: Acrylonitrile 69
CRRT: Continuous renal replacement therapy
ECMO: Extracorporeal membrane oxygenation
PK: Pharmacokinetics
